# LincRNA-MSTRG.673.2 Promotes Chicken Intramuscular Adipocyte Differentiation by Sponging miR-128-3p

**DOI:** 10.3390/ani15131879

**Published:** 2025-06-25

**Authors:** Binbin Zhang, Shuaipeng Zhu, Yuehua He, Wenjie Liang, Tingqi Zhu, Ruili Han, Donghua Li, Yanbin Wang, Yadong Tian, Guoxi Li, Xiangtao Kang, Wenting Li, Guirong Sun

**Affiliations:** 1 College of Animal Science and Technology, Henan Agricultural University, Zhengzhou 450046, China; zhangbinbin0711@163.com (B.Z.); zhu15039087513@126.com (S.Z.); 18438405655@163.com (Y.H.); wjliang2023@126.com (W.L.); ztq560713@163.com (T.Z.); rlhan@126.com (R.H.); lidonghua6656@126.com (D.L.); ybwang2008@126.com (Y.W.); ydtian111@163.com (Y.T.); liguoxi0914@126.com (G.L.); xtkang2001@263.net (X.K.); 2 The Shennong Seed Industry Laboratory, Zhengzhou 450002, China

**Keywords:** LincRNA-MSTRG.673.2, miR-128-3p, chicken, intramuscular adipocytes, differentiation

## Abstract

**Background:** Intramuscular fat content is positively correlated with meat flavor and juiciness. Increasing the intramuscular fat (IMF) content of chickens while increasing their growth rate has become a hot topic in molecular breeding. The group’s previous studies showed that miR-128-3p inhibited chicken intramuscular adipocyte differentiation and lipogenesis. However, the regulatory mechanism of miR-128-3p in intramuscular preadipocytes is currently unknown. In this study, we investigated the mechanism of miR-128-3p regulation of chicken intramuscular adipocyte differentiation and deposition. **Results:** Transcriptome data analysis of differential LincRNAs indicated that, compared to the NC group, the mimics-treated group had seventeen significantly differentially expressed LincRNAs (*p* < 0.05), including six upregulated and eleven downregulated ones; the inhibitor-treated group had seventeen differentially expressed LincRNAs (*p* < 0.05), including eight upregulated and nine downregulated ones; and twenty-four differentially expressed LincRNAs (*p* < 0.05) were observed when comparing the mimics-treated group to the inhibitor-treated group, with fourteen upregulated and ten downregulated ones. Functional enrichment analysis revealed that DELincRNAs from the overexpression group (M group) and interference group (SI group) were involved in the negative regulation of metabolic processes, response to steroid hormones, and regulation of actin cytoskeleton. Furthermore, target gene prediction analysis showed that miR-128-3p can target many of the DELincRNAs, such as LincRNA-MSTRG.673.2, LincRNA-MSTRG.39.2, LincRNA-MSTRG.39.3, and LincRNA-MSTRG.14270.2. LincRNA-MSTRG.673.2 was predominantly expressed in the cytoplasm of intramuscular adipocytes. Dual luciferase reporter identified the targeting relationship between miR-128-3p and LincRNA-MSTRG.673.2. The results of subsequent functional assays demonstrated that interfering with MSTRG.673.2 has been shown to inhibit lipid deposition in intramuscular preadipocytes. Transfection experiments have shown that LincRNA-MSTRG.673.2 can affect the expression of miR-128-3p. **Conclusions:** This study found that LincRNA-MSTRG.673.2 promoted chicken intramuscular adipocyte differentiation by downregulating miR-128-3p. The results are noteworthy for improving chicken meat quality, molecular breeding, and lipid metabolism research.

## Simple Summary

Our laboratory previously found that miR-128-3p inhibits the differentiation of chicken intramuscular adipocytes, but its regulatory mechanism remains unclear. In this study, transcriptome data analysis revealed 24 differentially expressed LincRNAs between the miR-128-3p mimics-treated group and the inhibitor-treated group, which were functionally enriched in metabolic regulation, hormone response, and other processes. Among them, LincRNA-MSTRG.673.2 was localized in the cytoplasm, and dual-luciferase reporter assays confirmed its targeting relationship with miR-128-3p. Functional experiments showed that interfering with MSTRG.673.2 inhibited lipid deposition, and this LincRNA could downregulate miR-128-3p expression. Therefore, we conclude that LincRNA-MSTRG.673.2 promotes intramuscular adipocyte differentiation by downregulating miR-128-3p. This study provides a new target for improving chicken meat quality and lipid metabolism research.

## 1. Introduction

Chicken is currently a major player in the market, with consumers increasingly demanding better taste. In chicken production, intramuscular fat is a crucial factor that positively impacts the quality and flavor of the meat [1]. The amount of intramuscular fat is determined by the capacity of intramuscular adipocytes to synthesize and store lipids [2]. Numerous research studies have unveiled a plethora of molecular mechanisms that control the differentiation of intramuscular adipocytes. These mechanisms include transcription factors [3,4], cell cycle regulators, non-coding RNA, signaling pathways [5] and others.

Micro-RNAs (miRNAs), a subclass of small non-coding RNAs with an 18–25 nucleotide length, are essential regulators of many biological processes, including cell division, proliferation, differentiation, and apoptosis [6]. For example, miR-425-5p [7] and miR-125a-5p [8] inhibit differentiation in porcine intramuscular preadipocytes. bta-miR-210 positively regulates the adipogenesis of PDGFRα + cells in bovine [9]. miR-340-5p inhibits sheep adipocyte differentiation. miR-223, gga-miRNA-18b-3p and miR-24-3p inhibit intramuscular adipocyte differentiation, while miR-15a and gga-miR-140-5p significantly promoted intramuscular adipogenic differentiation. miR-128-3p is involved in many physiological processes, including follicular development, gastric cancer cell development [10,11], and lung cancer cell development [12]. In addition, it is also a biomarker for detecting breast cancer in high-risk benign breast tumors [13], the diagnosis of non-small cell lung cancer [14], and lymphoblastic leukemia [15]. This suggests that miR-128-3p plays an important role in the regulation of various physiological and pathological processes in animals. miR-128-3p promotes GC apoptosis through 14-3-3β/FoxO pathway via repressing YWHAB and inhibits lipid synthesis by impeding the PPAR-γ/LPL pathway, as well as reducing the secretion of progesterone and estrogen [10]. And it impeded 3T3-L1 adipogenesis by targeting Pparg and Sertad2, resulting in the obstruction of preadipocyte differentiation and promotion of lipolysis [16]. Our previous research also showed that miR-128-3p inhibits intramuscular adipocyte differentiation in chickens by downregulating *FDPS* [17]. Therefore, we speculated that miR-128-3p might be a core miRNA regulated by intramuscular fat differentiation. Similar to miRNAs, long non-coding RNAs (lncRNAs) have also been found to regulate the transcription and translation processes of genes by interacting with DNA, RNA, or proteins. For example, LncRNA PVT1 facilitates the tumorigenesis and progression of glioma via the regulation of the MiR-128-3p/GREM1 axis and the BMP signaling pathway [18]. As intergenic lncRNAs, lincRNAs (long intergenic non-coding RNAs) have recently been demonstrated to play roles in adipose deposition: METTL14 modulates m6A methylation of LINC00278, which then binds to BRG1 to activate the PPAR-γ2 pathway and promote adipogenesis [19]; the knockdown of linc-ADAIN enhances the stability and translation of KLF5 and interleukin-8 (IL-8) mRNAs via interaction with IGF2BP2, leading to enhanced adipogenic programs and adipose tissue inflammation [20]; and LincRNA-ROFM has been shown to inhibit adipocyte proliferation and differentiation. Collectively, these findings indicate that lincRNAs can exert regulatory effects on adipose deposition [21].

In this study, we identified several key LincRNAs that play a role in the regulation of lipid metabolism pathways via miR-128-3p using transcriptome data and functional prediction. We explored the interaction between LincRNA-MSTRG.673.2 and miR-128-3p. In addition, the results of our cell functional assays confirmed that LincRNA-MSTRG.673.2 regulates the differentiation of intramuscular adipocytes in chickens. This will provide new ideas for a better understanding of the molecular mechanisms of chicken intramuscular adipocyte differentiation and new strategies and methods for improving chicken meat quality.

## 2. Materials and Methods

### 2.1. Collection of Sequencing Samples

The transfected miR-128-3p-mimics, miR-128-3p-inhibitor, and miR-128-3p-NC were divided into overexpression group (M group), interference group (SI group), and blank treatment group (NC group). The overexpression and interference fragments of miR-128-3p were synthesized by Shanghai GenePharma Co., Ltd. (Shanghai, China). The sequence information is as follows: gga-miR-128-3p-mimics (UCACAGUGAACCGGUCUCUUU) and gga-miR-128-3p-inhibitor (AAAGAGACCGGUUCACUGUGA). The expression of miR-128-3p was detected by Q-PCR. Subsequently, samples with high overexpression efficiency (*n* = 3), high interference efficiency (*n* = 3), and samples from the blank group were sent to Nanjing Parsono Gene Technology Co., Ltd. (Nanjing, China) for transcriptome sequencing. The overexpression and interference efficiency assays for the sequencing samples are illustrated in Supplementary Figure S1.

### 2.2. Transcriptome Data Analysis

After the removal of raw reads containing no insertion sequence, over 0.2% of poly-N, and low-quality paired reads, we obtained clean reads. The high-quality data (clean data) were mapped to the reference genome (GRCg7a) using TopHat2’s upgraded HISAT2 software (version 2.2.1) [22]. Read count values were aligned to each gene using HTSeq statistics as the gene’s original expression quantity [23]. CPC [24], CNCI [25], and PFAM [26] were used to distinguish the protein-coding genes from the noncoding genes. Differential LincRNAs expression analysis was conducted using DESeq [27], and differentially expressed LincRNAs were defined based on the following criteria: |log_2_ fold change| > 1 and the *p* value < 0.05. Gene ontology (GO) [28] and (KEGG) [29] analysis were used for predicting the function of the DE lncRNAs.

### 2.3. Primary Intramuscular Adipocyte Isolation and Culture and Induced Differentiation

Intramuscular adipocytes were isolated as previously described [30]. Breast muscle tissue was collected from 14-day-old Gushi chicken under sterile conditions and then digested using 2 mg/mL collagenase type II (Sangon Biotech, Shanghai, China) with shaking for 2 h at 37 °C. Then, the tissue was cut into 1 mm^3^ pieces using surgical scissors and then digested by adding appropriate amounts of collagenase type I (Solaibao, Beijing, China) with shaking for 1 h at 37 °C. The digested cell suspension was filtered through 45 and 75 mum filters collected by centrifugation at 1000 r/min for 10 min. Subsequently, the cells were kept in DMEM/F12 media (Business Intelligence (BI), Boston, MA, USA) supplemented with 10% fetal bovine serum (Business Intelligence (BI), Boston, MA, USA) and 1% penicillin/streptomycin (Solarbio, Beijing, China) in an incubator that maintained a 5% CO_2_ environment at 37 °C. Cells were cultured for 2 h, followed by a fluid change. Once the intramuscular adipocyte confluence reached 90%, the media was replaced entirely by the differentiation-inducing media composed of 0.5 mM of 3-isobutyl-1-methylxanthine (IBMX), 1 uM of dexamethasone (Sigma, St. Louis, MO, USA), and 10 g/l of insulin (Sigma) [31]. Pentobarbital was used for euthanization by intraperitoneal injection at a dose of 40 mg/kg of body weight.

### 2.4. Transient Transfection of Intramuscular Adipocytes

Cells were transfected with miR-128-3p mimics, inhibitors, and LincRNA-MSTRG.673.2-Si with Liposome 3000 reagent (Invitrogen, Waltham, MA, USA) according to the manufacturer’s instructions. The working concentration of miR-128-3p mimics, inhibitors, and LincRNA-MSTRG.673.2-Si was 20 pmol/mL for all. Then, the medium was changed to a complete medium after 6 h, and the interference efficiency was evaluated with qRT-PCR after 2 d.

### 2.5. Localization Analysis of LincRNA-MSTRG.673.2 Using Fluorescence In Situ Hybridization (FISH) and Nuclear/Cytoplasmic RNA Separation

Fluorescence in situ hybridization was designed and synthesized by Wuhan Saviour Biotech Co., Ltd. (Wuhan, China). The probe information was as follows: 5′-3′ GAGGAACAGGAGAGATATGCTACTCATT-TTGTATA. The intramuscular fat cells were attached to the glass slide and grew to 80% density. The operation was carried out according to the instructions of the in situ hybridization kit (Wuhan Saviour Biotech Co., Ltd.). Four-percent paraformaldehyde was used to fix the cells, DAPI was used to stain the cells, and images were captured under a fluorescence microscope. CY3 red light had an excitation wavelength of 510–560 nm and an emission wavelength of 590 nm, emitting red light. The primary intramuscular preadipocytes that were cultured were digested using trypsin, and the digestion was terminated with complete culture medium. Subsequently, PBS was added for preservation, and the subsequent operations were carried out according to the nuclear–cytoplasmic separation kit. The collected cytoplasmic mixture and cytoplasmic precipitate were separately subjected to RNA extraction using the Trizol method. Then, using U6 as the internal reference, the expression levels of MSTRG.673.2 in the cytoplasm and nucleus were quantitatively detected.

### 2.6. Dual Luciferase Reporter Assay

The laboratory psiCHECK2 vector was used to construct the psiCHECK2-LincRNA-MSTRG.673.2-WT and psiCHECK2-LincRNA-MSTRG.673.2-MuT plasmids. They were transfected with miRNA-128-3p-mimics and psiCHECK2 vector into DF1 cells. After 48 h, the samples were collected, and the fluorescence activity was detected using the Dual-Glo Luciferase Assay Systemt (Promega, Madison, WI, USA).

### 2.7. Oil-Red O Staining Triglyceride Assay Detection of Cell Differentiation

The intramuscular adipocyte samples were fixed with 4% paraformaldehyde [16], and Oil Red O working solution (Sigma) was used for cell staining. Intramuscular fat cells were photographed under a microscope (Nikon, Tokyo, Japan). After microscopic examination, isopropanol was used to dissolve the lipid droplets, and the absorbance value was calculated at 490 nm. The triglyceride content in the cell homogenate was determined using a triglyceride content detection kit (APPLYGEN, Beijing, China). The protein concentration was measured using a BCA protein assay kit (EpiZyme, Shanghai, China) to standardize both the triglyceride content and the results of Oil Red O staining. The absorbance values were calculated at 550 nm using a microplate reader.

### 2.8. Q-PCR

Total RNA was extracted from intramuscular adipocytes using Trizol (Vazyme, Nanjing, China). mRNA was reverse transcribed using the HiScript II Q Select RT SuperMix for qPCR kit (Vazyme, Nanjing, China), and quantitative real-time PCR was performed using the ChamQ Universal SYBR qPCR Master Mix kit (Vazyme, Nanjing, China). Due to the differences in reverse transcription systems between miRNA and mRNA, the specific reverse transcription procedures for miRNA are described here. The first-step reaction program is as follows: 42 °C for 2 min, 2 μL of 5× gDNA wiper mix, 1 μg of total RNA, with a total reaction volume of 10 μL. The second-step reaction program is 37 °C for 15 min, 85 °C for 5 s, using 10 μL of the first-step reaction product, 2 μL of 10× RT mix, 2 μL of HiScript III Enzyme Mix, 0.5 μL of miRNA RT primer, 0.5 μL of U6 RT primer, and 5 μL of RNase-free ddH_2_O. The relative expression levels were calculated using the 2^−ΔΔCt^ method [32]. The primer sequences are shown in Supplementary Table S1.

### 2.9. Statistical Analysis

Data analysis was performed with SPSS 26.0. All data was presented as “mean ± standard error (SEM)”. Significant differences between groups were analyzed using one-way ANOVA. Asterisks signify different significance levels (# *p* > 0.05, * *p* < 0.05, ** *p* < 0.01, and *** *p* < 0.001).

## 3. Result

### 3.1. Identification and Characterization of LincRNAs in Chicken Intramuscular Adipocyte Groups Post miR-128-3p Overexpression and Interference

**Figure 1:**
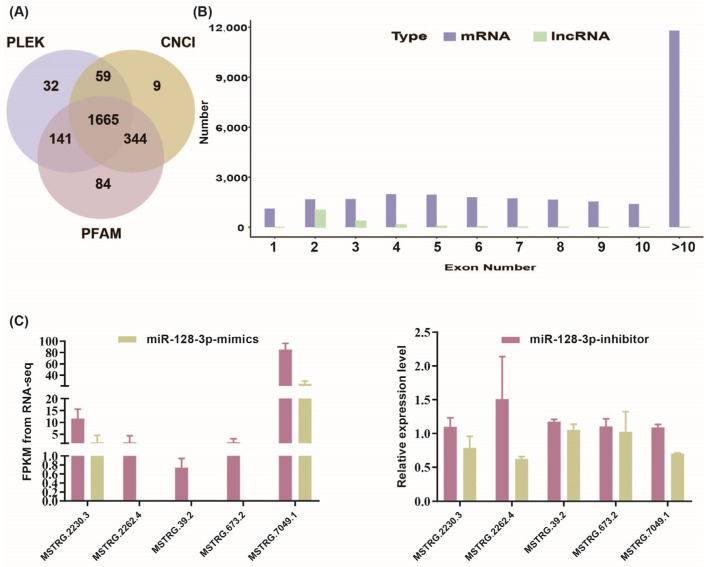
Characterization of LincRNAs. (**A**) Venn diagram of LincRNAs predicted by CNCI, CPC2, PLEK, and PFAM. (**B**) Exon number distribution of mRNAs and lincRNAs. (**C**) Validation of LincRNA sequencing data.

To identify the major LincRNAs that interacted with miR-128-3p and involved in the regulation of intramuscular fat deposition. A model of miR-128-3p differentiation in chicken intramuscular adipocytes was constructed, including mock-treated and inhibitor-treated groups, and transcriptome sequencing was performed. After additional filtering and removal of potential coding transcripts that were identified using CNCI, CPC, PFAM, and CPAT (Figure 1A), a total of 1665 lnicRNAs were obtained. Comparative analyses of gene structure, expression, and sequence conservation were used to explore differences between LincRNAs and protein codes genes. lncRNAs were typically shorter than mRNAs (Figure 1B). lncRNAs also appeared to be expressed at lower levels than mRNAs. This suggested that the LincRNA obtained by sequencing conformed to the characteristics of a non-coding RNA. To confirm the reliability of the RNA-seq results, 5 DELincRNAs (MSTRG.2230.3, MSTRG.2262.4, MSTRG.39.2, MSTRG.673.2, MSTRG.7049.1) were randomly selected, and their expression levels were measured by qPCR (Figure 1C). As expected, the expression levels of all 5 candidate LincRNAs showed a consistent trend of expression, therefore validating our results.

### 3.2. Analysis of Differential LincRNAs Expression and Screening for miR-128-3p Targeting LincRNAs

**Figure 2:**
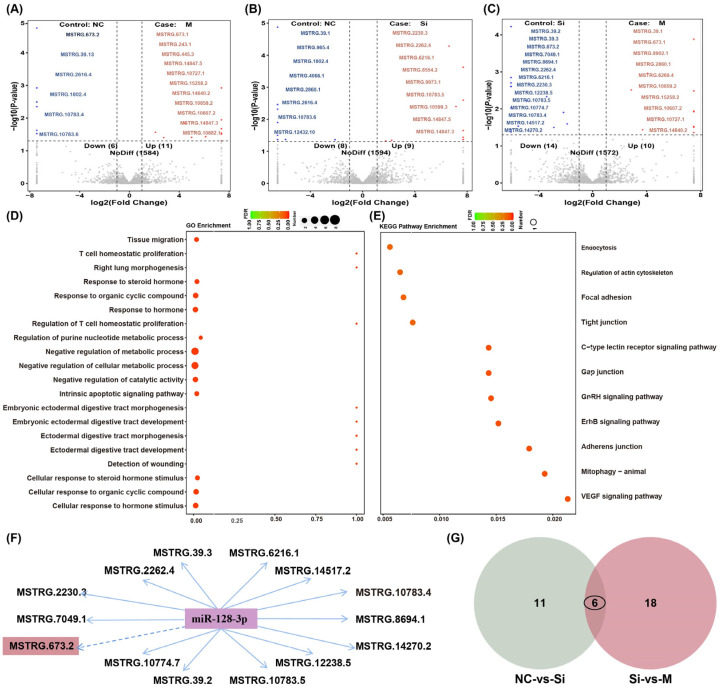
Differential LincRNAs expression analysis. (**A**–**C**) Volcano plot of gene expression in the NC vs. M, NC vs. SI, and SI vs. M comparisons. Numbers of upregulated and downregulated differentially expressed LincRNAs. The left blue bars represent the numbers of upregulated genes; the orange bars represent the numbers of downregulated genes. (**D**) GO pathway enrichment analysis of the DELincRNAs in the M vs. SI comparison. (**E**) KEGG pathway enrichment analysis of the DELincRNAs in the M vs. SI comparison. (**F**) Based on the comparison of binding sites in the seed region of miR-128-3p, Cytoscape software was used for network interaction analysis, and the regulatory network of miR-128-3p was mapped. (**G**) Venn diagrams of DELincRNAs identified by RNA-seq in the NC vs. M, NC vs. SI, and SI vs. M comparisons.

Transcriptome data analysis of differential LincRNAs indicated that, compared to the NC group, the mimics-treated group had seventeen significantly differentially expressed LincRNAs (*p* < 0.05), including six upregulated and eleven downregulated ones (Figure 2A); the inhibitor-treated group had seventeen differentially expressed LincRNAs (*p* < 0.05), including eight upregulated and nine downregulated ones (Figure 2B); and twenty-four differentially expressed LincRNAs (*p* < 0.05) were observed when comparing the mimics-treated group to the inhibitor-treated group, with fourteen upregulated and ten downregulated ones (Figure 2C). GO function enrichment analysis revealed that DELincRNAs from the overexpression group (M group) and interference group (SI group) were involved in the negative regulation of metabolic processes, response to steroid hormones, and negative regulation of cellular metabolic processes (Figure 2D). KEGG pathway analysis showed that DELincRNAs were involved in the regulation of the actin cytoskeleton, focal adhesion, tight junctions, and the VEGF signaling pathway (Figure 2E). Upregulated miRNAs affect fat accumulation by downregulating their target LincRNAs. The differential LincRNAs potentially regulated by miR-128-3p, as obtained from the transcriptome data, were compared with the binding sites in the seed region of miR-128-3p. Subsequently, Cytoscape software (Version 3.9.1) was used to construct a network of targeting relationships centered on miR-128-3p. In this network, miR-128-3p can target 14 downregulated LincRNAs, such as MSTRG.673.2, MSTRG.2262.4, MSTRG.6216.1, and MSTRG.8694.1 (Figure 2F). To increase the probability that the selected LincRNAs regulate IMF deposition, we investigated whether these potential target LincRNAs overlapped among the two comparisons (NC vs. SI and SI vs. M). miR-128-3p were targeted by six common LincRNAs (Figure 2G).

### 3.3. LincRNA-MSTRG.673.2 Targets Binding to miR-128-3p

**Figure 3:**
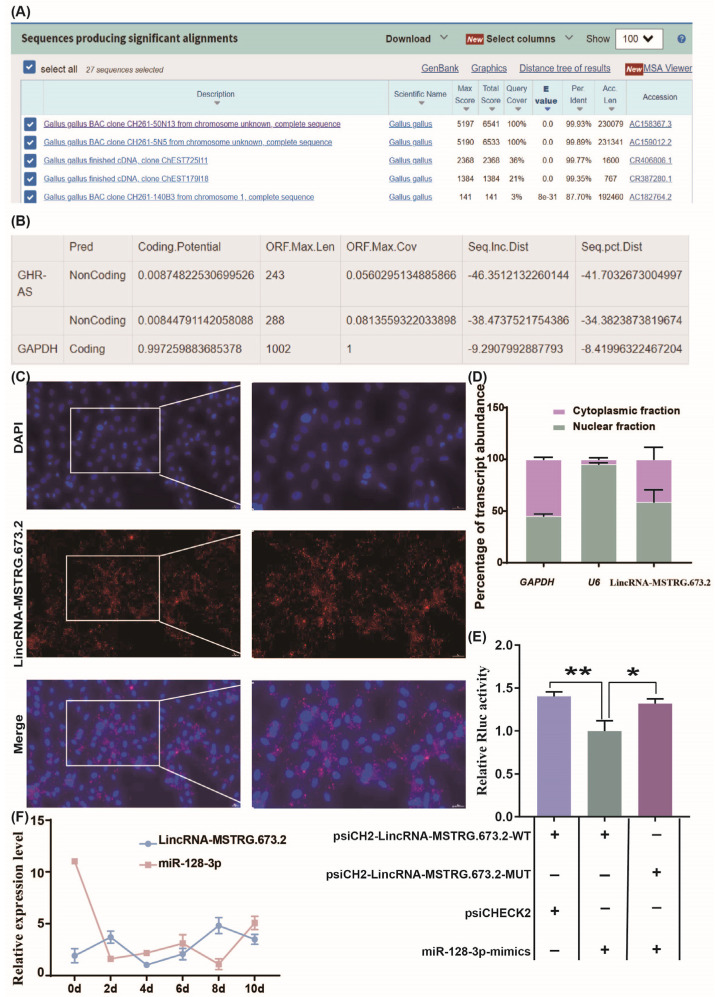
LincRNA-MSTRG.673.2 targets binding to miR-128-3p (**A**,**B**). LincRNA-MSTRG.673.2 coding ability evaluation. (**C**) LincRNA-MSTRG.673.2 in situ hybridization analysis. (magnification 200). (**D**) Cytoplasm and nucleus RNA were extracted from intramuscular preadipocytes. LincRNA-MSTRG.673.2 cellular location was studied by qRT-PCR assay. (**E**) The DF1 cells were cotransfected with either psiCHECK2-LincRNA-MSTRG.673.2-WT or psiCHECK2-LincRNA-MSTRG.673.2-MuT and miR-128-3p-mimics or psiCHECK2. Then, the relative luciferase activity was measured. * *p* < 0.05, ** *p* < 0.01. (**F**) The expression levels of LincRNA-MSTRG.673.2 during the differentiation of chicken primary intramuscular adipocytes into mature adipocytes.

According to the online BLAST analysis conducted on the NCBI website, it was found that LincRNA-MSTRG.673.2 belongs to the non-coding sequence and does not overlap with any known genes (Figure 3A). Further inquiry on the LncFinder website revealed that LincRNA-MSTRG.673.2 shares similar non-coding RNA coding characteristics with the known LncRNA-GHR-AS. The coding ability scores of both are much lower than the commonly used reference gene GAPDH, indicating that they are non-coding sequences (Figure 3B). RNA FISH indicated that LincRNA-MSTRG.673.2 is predominantly localized in the cytoplasm of preadipocytes (Figure 3C). After separately extracting cytoplasmic RNA and nuclear RNA from intramuscular preadipocytes, the relative expression level of MSTRG.673.2 was analyzed. The results showed that MSTRG.673.2 was localized in both the nucleus and cytoplasm (Figure 3D). We used online software to evaluate the protein coding ability of LincRNA-MSTRG.673.2. Analysis of the sequence was conducted by online software NCBI-BLAST (version 2.13.0+). Using a dual-luciferase reporter assay, we further validated whether LincRNA-MSTRG.673.2 served as ceRNA of miR-128-3p. Compared to the cotransfection of psiCHECK2-LincRNA-MSTRG.673.2-WT and miR-128-3p-mimics groups, the luciferase activity was significantly upregulated in the cotransfection of psiCHECK2 and psiCHECK2-LincRNA-MSTRG.673.2-WT groups (*p* < 0.01). Similarly, the cotransfection of psiCHECK2-LincRNA-MSTRG.673.2-MuT and miR-128-3p-mimics groups also showed a significant increase compared to the control group (*p* < 0.05). This indicated the targeted binding relationship between LincRNA-MSTRG.673.2 and miR-128-3p (Figure 3E). To further investigate the regulatory effects of LincRNA-MSTRG.673.2 and miR-128-3p on intramuscular fat deposition in chickens, quantitative detection was used to examine the expression of miR-128-3p and LincRNA-MSTRG.673.2 at different time points (0, 2, 4, 6, 8, and 10 days) after induction of intramuscular fat differentiation. The results showed that LincRNA-MSTRG.673.2 and miR-128-3p exhibited opposite expression patterns during intramuscular adipocyte differentiation (Figure 3F).

### 3.4. Interfering with LincRNA-MSTRG.673.2 Inhibits Lipid Deposition in Intramuscular Preadipocytes

**Figure 4:**
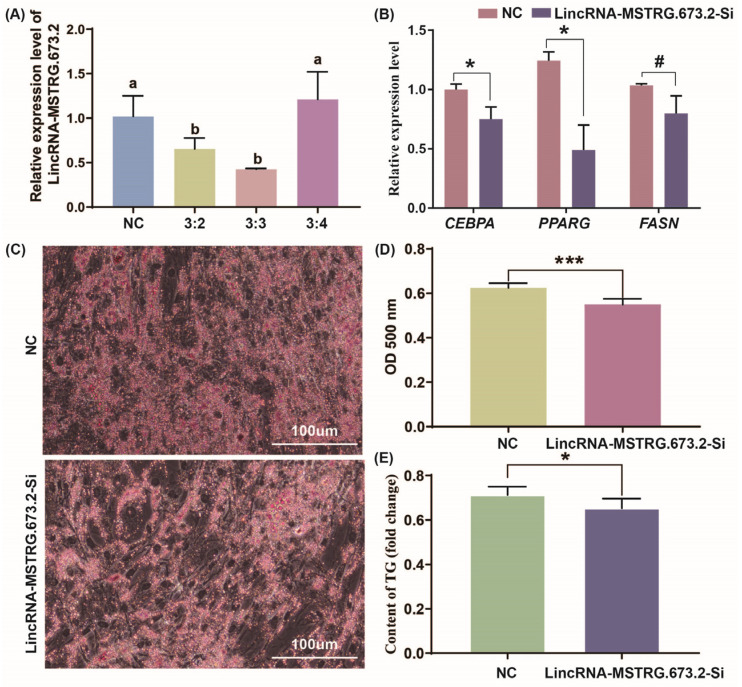
Interference with LincRNA-MSTRG.673.2 expression inhibits intramuscular preadipocyte differentiation. # *p* > 0.05, * *p* < 0.05, and *** *p* < 0.001. (**A**) Detection of LincRNA-MSTRG.673.2 interference efficiency. (Different letters indicate *p* < 0.05; 3:2 indicates the volume ratio of Lip3000 to the diluted transfection fragment is 3 μL:2 μL.) (**B**) Relative mRNA levels of adipocyte differentiation-related genes (*CEBPA*, *PPARG*, and *FASN*) after interference with LincRNA-MSTRG.673.2 expression. (**C**) Representative images of Oil Red O staining in intramuscular adipocytes transfected with LincRNA-MSTRG.673.2 siRNA or the corresponding NC. (**D**) Semiquantitative assessment of Oil Red O absorbance at 450 nm. (**E**) The triglyceride content was determined by measurement of the absorbance at 500 nm after interference with LincRNA-MSTRG.673.2 expression.

To ascertain the effect of LincRNA-MSTRG.673.2 in intramuscular preadipocyte differentiation, LincRNA-MSTRG.673.2 inhibitor or NC was transfected into chicken intramuscular preadipocytes, respectively. The results showed that the interference effect could reach over 50%, meeting the experimental requirements (Figure 4A). The expression of adipogenic genes (*CEBPA*, *PPARG*, *FASN*) were remarkably reduced in LincRNA-MSTRG.673.2 treated cells (Figure 4B). Subsequently, Oil O red staining and intracellular triglyceride content detection were performed. The Oil O red staining showed the lipid droplet accumulation in the LincRNA-MSTRG.673.2 interference group was significantly lower than that in the blank control group (*p* < 0.01) (Figure 4C,D). The intracellular triglyceride content detection indicated that that interfering with LincRNA-MSTRG.673.2 decreased intracellular triglycerides (*p* < 0.05) (Figure 4E). These results suggest that interfering with LincRNA-MSTRG.673.2 reduced lipid deposition in intramuscular preadipocytes, thereby achieving an inhibitory effect on differentiation.

### 3.5. LincRNA-MSTRG.673.2 Promoted Intramuscular Differentiation by Adsorbing miR-128-3p

**Figure 5:**
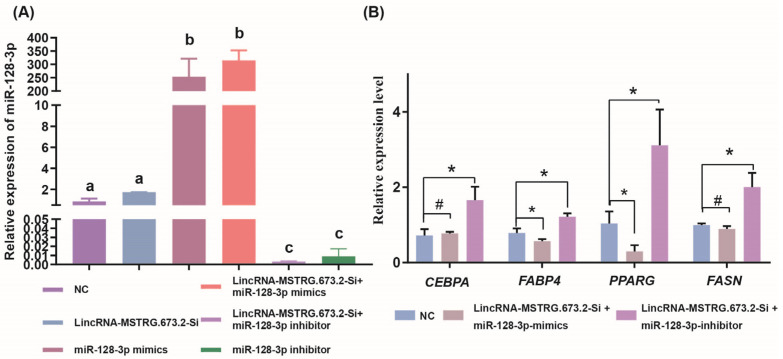
LincRNA-MSTRG.673.2 promoted intramuscular differentiation by adsorbing miR-128-3p. (**A**) Effects of cotransfection LincRNA-MSTRG.673.2-Si and miR-128-3p-mimics/inhibitor on the expression of miR-128-3p. (Different letters indicate *p* < 0.05.) (**B**) Effect of cotransfection MSTRG.673.2-Si and miR-128-3p-mimics/inhibitor on the expression of adipose differentiation marker genes. # *p* > 0.05, * *p* < 0.05.

After cotransfection of LincRNA-MSTRG.673.2-Si with miR-128-3p-mimics/inhibitor, the expression levels of miR-128-3p was measured. Compared to the blank control group, the expression level of miR-128-3p was significantly upregulated after interfering with LincRNA-MSTRG.673.2 (*p* < 0.05). Simultaneously interfering with LincRNA-MSTRG.673.2 and overexpressing miR-128-3p led to a highly significant upregulation of miR-128-3p expression (*p* < 0.01). Furthermore, when interfering with both LincRNA-MSTRG.673.2 and miR-128-3p, the expression level of miR-128-3p was significantly downregulated (*p* < 0.01) (Figure 5A). This indicated that LincRNA-MSTRG.673.2 had an inhibitory effect on the expression of miR-128-3p. Additionally, compared with the blank control group, interfering with LincRNA-MSTRG.673.2 and simultaneously overexpressing miR-128-3p resulted in a significant downregulation of adipogenic marker genes such as *FABP4* and *PPARG* (*p* < 0.05). When interfering with both LincRNA-MSTRG.673.2 and miR-128-3p, *CEBPA*, *FABP4*, *PPARG*, and *FASN* were significantly upregulated (*p* < 0.05) (Figure 5B).

## 4. Discussion

Adipose tissue, with its highly active secretory capacity, can absorb or release non-coding RNAs, leading to a significant role for miRNAs and lncRNAs in the process of fat deposition [33,34]. In this experiment, we specifically selected samples of cells overexpressing/underexpressing miR-128-3p for transcriptome sequencing. We identified 14 upregulated and 10 downregulated differential LincRNAs. Functional enrichment analysis revealed that DELincRNAs were involved in the regulation of the actin cytoskeleton, focal adhesion, tight junctions, and the VEGF signaling pathway. A series of pathways related to cell junctions, including tight junctions, focal adhesion, and the regulation of the actin cytoskeleton, might contribute to the deposition of IMF [35]. Fatty acid metabolism-related candidate genes also were involved in these pathways. VEGF inactivation may induce fat accumulation [36]. In addition, some studies have shown that VEGF inactivation induces fat accumulation. Through analysis of significantly differentially expressed LincRNAs following interference, we identified LincRNAs regulated by miR-128-3p, such as LincRNA-MSTRG.673.2, LincRNA-MSTRG.39.2, LincRNA-MSTRG.39.3, and LincRNA-MSTRG.14270.2. Subsequently, by comparing the lengths of these lncRNAs and their binding strength with miR-128-3p, we selected LincRNA-MSTRG.673.2 as the subject for subsequent research.

The specific mechanisms by which lncRNAs regulate miRNAs include acting as potential pri-miRNAs to generate mature miRNAs, indirectly regulating target gene expression [37]; indirectly inhibiting miRNA-mediated negative regulation of target genes by competitively binding to the 3′-UTR of miRNA target mRNAs [38]; and serving as a “miRNA sponge” to inhibit miRNA expression [39]. On one hand, miRNAs can bind to the 3′-UTR region of lncRNAs, thereby reducing the stability and expression of lncRNAs [40]. On the other hand, miRNAs can return to the cell nucleus and regulate the transcription of lncRNAs, enhancing lncRNA expression. Long noncoding RNA HCP5 contributes to nasopharyngeal carcinoma progression by targeting microRNA-128-3p [41]. The knockdown of LncRNA DLEU2 inhibits cervical cancer progression via targeting miR-128-3p [42]. In this study, LincRNA-MSTRG.673.2 was mainly localized in the cytoplasm. This study analyzed and verified the existence of a targeting relationship between LincRNA-MSTRG.673.2 and miR-128-3p. Also, LincRNA-MSTRG.673.2 and miR-128-3p exhibited opposite expression patterns during intramuscular adipocyte differentiation. Hence, the LincRNA-MSTRG.673.2 acted as a sponge for miR-128-3p. lncRNAs are involved in a variety of bioregulatory processes, including muscle growth, fat deposition, and lipid metabolism [43]. For example, lncSAMM50 [44] and lncRNA [45] enhance adipocyte differentiation. We showed that interfering with LincRNA-MSTRG.673.2 inhibited intramuscular fat deposition. The group’s previous research showed that miR-128-3p could inhibit the differentiation of intramuscular adipocytes, a result consistent with the interference of LincRNA-MSTRG.673.2. Additionally, after interfering with LincRNA-MSTRG.673.2, miR-128-3p was significantly upregulated, aligning with the mechanism of action between LincRNA and miRNA. For example, LincRNA TINCR promoted lipid deposition by absorbing miR-31-5p to enhance the expression of CEBP pathway genes [46]. *PPARG* is indeed a direct target of miR-128-3p [47]. Transfection experiments with LincRNA-MSTRG.673.2-Si and miR-128-3p-mimics showed that interfering with LincRNA-MSTRG.673.2 upregulated the expression of miR-128-3p and downregulated the expression of adipocyte differentiation marker genes (*CEBPA*, *FABP4*, *PPARG*, *FASN*). This implied that lncRNA may have competitively adsorbed miR-128-3p, thereby affecting *PPARG* and potentially influencing intramuscular fat deposition. Our research identified LincRNA-MSTRG.673.2 targeting miR-128-3p to promote chicken intramuscular adipocyte differentiation. Determining miR-128-3p as a key regulatory factor for intramuscular preadipocyte fat deposition in chickens is of significant importance for breeding practices aimed at improving chicken meat quality.

## 5. Conclusions

In conclusion, this study demonstrated that LincRNA-MSTRG.673.2 could promote chicken intramuscular adipocyte differentiation by sponging miR-128-3p. These findings may contribute to furnishing new insights for improving chicken quality breeding.

## Data Availability

The RNA sequencing data used and analyzed during the current study are available from the NCBI (accession number: PRJNA986221).

## Supplementary Materials

The following supporting information can be downloaded at: https://www.mdpi.com/article/10.3390/ani15131879/s1, Additional File S1: Table S1: Q-PCR primer sequences. Additional File S2: Figure S1: Overexpression and interference efficiency detection of sequencing samples.

## Abbreviations

*CNCI*	Coding–noncoding index
*CPC*	Coding potential calculator
*IMF*	Intramuscular fat
*MiRNA*	MicroRNA
*NC group*	Blank treatment group
*M group*	Mimics-treated group
*SI group*	Inhibitor-treated group
*GO*	Gene ontology
*KEGG*	Kyoto encyclopedia of genes and genomes
*Q-PCR*	Quantitative PCR
*PBS*	Phosphate-buffered saline
*PPARG*	Peroxisome proliferator-activated receptor γ
*FABP*	Fatty acid binding protein 4
*FASN*	Fatty acid synthetase
*CEBPA*	CCAAT/enhancer-binding protein alpha
*FDPS*	Farnesyl diphosphate synthase
