# Supplementary material for: LincRNA-MSTRG.673.2 Promotes Chicken Intramuscular Adipocyte Differentiation by Sponging miR-128-3p
Source: Animals (Basel). 2025 Jun 25;15(13):1879. doi: 10.3390/ani15131879 (PMC12249141; doi:10.3390/ani15131879)

Table S1. q-PCR primer sequences

Gene	Reverse primer sequence (5'-3')	Application
miR-128-3p	GTCGTATCCAGTGCAGGGTCCGA<br>GGTATTCGCACTGGATACGACGGCCGG	RT-PCR
	F: CGCGCGCGCACAGGAA<br>R: AGTGCAGGGTCCGAGGTATT	q-PCR
U6	GTCGTATCCAGTGCAGGGTCCGA<br>GGTATTCGCACTGGATACGACCGATACA	RT-PCR
	F: GGGCCATGCTAATCTTCTCTGTATCG<br>R: GTGCAGGGTCCGAGGT	q-PCR
β-actin	F: CAGCCAGCCATGGATGATGA<br>R: ACCAACCATCACACCCTGAT	q-PCR
PPARA	F: AATGAAATTCAATGCACTGG<br>R: CAGTTTCAGTACATGCACA	q-PCR
CEBPA	F: AAGATGATGCCCGCCGACCAC<br>R: CATGGCCTTCACCAGCGAGCTT	q-PCR
FASN	F: GATTGTTTCCCCACTTCGAC<br>R: CTGTTGCCAGCATAATGTCC	q-PCR
PPARG	F: ACCTCACGAGGAGTCTTCCA<br>R: GCTTCTCCTTCTCCGCTTGT	q-PCR
FABP 4	F: CTGCTACCTGGCCTGACAAA<br>R: CAGTGTGCCACTGTCTAGGG	q-PCR
MSTRG.67<br>3.2	F: GAAACAATGACTGTAGCCA<br>R: CAGAAATATTGACATTCCGA	q-PCR
MSTRG.39.<br>2	F: CAACAAGAGCATCTCGGACCT<br>R: CTCCAGCTTCATCTGCCGGAA	q-PCR
MSTRG.70<br>49.1	F: CTGAGCTGCCATCAGGCACTG<br>R: AGCTCACACGTAGGACAAGGG	q-PCR
MSTRG.22<br>30.2	F: GATTCAGCAGTGGTGGTGCCTTC<br>R: ATCCTTGTACTGCATCCCC	q-PCR
MSTRG.22<br>62.4	F: CACTGCTCCTCTGATCCTTG<br>R: AGGCAATCAAATTTTAACTGGA	q-PCR

Abbreviation:F and R refer to the forward and reverse primer

Supplementary Sequences

AAATAAAACCACTTTACTGTTTAGAATAAAAGGACACTATAAAAAGTGAACATTATGCAACATTACCTTAAAAGACAATATACATTCACTGAATATAAAATTTATAAATAACATGGAGG
AAAACTGTAAACAGTGCTAGAAAATTTAGCAACAAATACATTCCTTCTGGACAAGATTTTTCACGCAGGTTTGGTTCCCTCCCCAAATTTCCATGACTATCAGGAACAGCAGAGTGGCTC
TGTGAAGGAAGGGTAGTGTCAGAGAGAATGGGAAACCAGTACAGGCAAAGCAGCAGTGCTTAGCAGAGTGGTAAGCTTGTTGTCAAGACTGGTAACACCAGAAGAAACTTATTCCAAT
TTAAAAAGGTTCTGCAAAAGTTTTGCCCTGCCTTTCTAAGCTAGAACAGACATTTTAAATATATTTTTCAAAGACATCTGAAAGAATGGAGGTTTGTTCCCATGGAGGCTTTTACTTCGAG
CCCTTACTGTGAAAAACACCGCAGTGTGTTTGCCATTAATCACCCTGAGATAGTACTCCAACCAGCATGAATGGAATATGGTGATACGTTTAGGTTTCCAAATAGAGCTCTGTAAAAGGC
CATGCAATTTCTCAAAACATGAGAAGTTTAGTCCTAAACTTGCAGTTTTGTCCCATGGAGGAGTGAGGGAAGCCTACCACAGAGGGGGACCGCAGTATCTCAGTGATCCCAAGAGGACT
TTAGTCAAGGTACTCCTAGCACAGGTCTCTTCCTGTTCTCTATCACTCCCCTTTTTGGAAAGAAAAATCGAGATCAATTATTCCCTACCCTTCCATATATTTTTAATACTTTTTTATAATTTC
TAAGGGGAAACACTCTCAAGCAAAGAAAGGGAAAATATAAATAATTATAATTTAAAAAGCAAGAGAATTTCTAATAATGTTGGTGTATAGAGGGGAAAATTGCTCTCATCCTACCTGTG
CCCAAAGATCATGCTCACATGAGCTGGGCAGTTGATAGTTTCCATCTGATCTTTGATTGCAGGGTAAAGTGAGGAGCATGGGAGAGGTGATTTGTGTGAGCCACTGCATGCTCCAGTA
CTCAGTATTTGGGGGAGAACTTCATACCTGCCTCCTCACATCCCATCTCTCAGGGCCCACATCCTTCATGGTGTGTTCTAACAACTGAGAACAATCATAGAATCACCTAGGTTGGAAA
AGACCTCCATGATCATCTAGTCCAACCATCCGTCTACCACCAATACTTCCCCACAAAACCGCGTCCCTTAGTACAACATCTAAATGTTTCTTGATTGCTTTGAGCTTTATCTAACAACAG
GCAGTAGTGACTTAGCGGGATAGGAGAAAGGAGAGCACAGAGAGAAAGGAGTAAATCTTAGAGTCTGAATTAGAGCCCATACAAGCCACGTTAACAGCTCCCTTCTACTTCAAGGGA
ACCTTCAGGCAGGTCCTTAATGCAAGCACAAGCTAAAGCTTAGCTCAAGAAGCACACGTCCCTGATAGCATGCAAAGAAACAAAATTAAGTAGCTTCCCAAAAGAAATGTATTTTTACT
GGATTCTTCCCCCTCCTTTTGGCTCCACATTTTTAAAAGTCTTAACAACTGTTTCTTTACACAACAAGGGAAACTATTTGCTACCAAACCCCAACCCTTGCTGGTCTAGCTGTATGCATC
TCAGAAATATTTACCGTCTGCAATTAAACTGTGAATTCAATTCATTTTCTGGTAATGTGGTTGCTGTTTTGTTGTTTACAAAGCACATAGACTCGTTATATCCTACTTGGGGACACAAGC
CCTCATAGCATTAGAATGAAAATATTTTCACAATAATTGGAGTGCAATACTGTGATGTCTCAGCTATAGCTACATGAATCAGTGACAGGTAAAATATGCAGAGCTGCAATTACATGAAAA
ATCAACACCTCACTGCTTCGAGGGGCATTCTCATTCAGCGTCCGAAGCCGTGATAAGTGGCTGCAGCTCAAAGAAACATCAGCTTTGCGAGGTCGCTTTGAGCTTGAGGAGCATCTC
CAGAACCGCTGACTCCACACAGTGTGGACAGTGAGGGTGGAAGCATCCCTGCTGGCCAGGCTGGCTCCACTCTGCACTCAACTGAGATTGAGCCCTCGGAGATCTTGTTGGCAGTG
CACAGCTCAAACAGAGGTTGCCCCACTGTGTCCAAAAGCCTGATCCCATCTCTGCCAAAAGGGGCAAAGCTCTTGACCTGAACCTATGATGGGTGCTACGAAGCACCCTACTCTCCC
TCTCATCTCCAACCTATAATTTAGGAGTAGTCACAGGGAAGAGGACTTTTTGAAAAGTGTTTCAAGCCTGAATCACAGGAAAATCCAATGGATTTTCAGTGTGTTGAGTTCTGTATTGC
CACTCAGAGCAGCTGGAAAAAAAAAATACACAGCAAACCTAGGTGCCTCCTATTCCAGCTCTTTGAAAATGCCCTTTAAAGCTCATGCTGAAAATGAAGCTGTAACTCTTTTTGCTGTA
ACAATTCACCTAGTGAGTGGGGCATTTCAAGGCATGTACATCTGCCAAAGCACTGGTAGCATAGAAGAGCCACAAAAATGCATCAGGACCCATCTCAGACATCCATTCCTTTCCATTT
AGCAAAGAGGCCCCAAACAACTCTCCAATAGTTGAGAAGGTCACTGACTATAAAACGCTGTATTGTCCTGGGCTGGATCGCTGCAACACTTTCTCCTGCTGGTGGCAGCTCTGCTTTC
TAAGTGATGAATGAGATATATGGAAACTAGATAATATCATAATATACAAAATGAGTAGCATATCTCTCCTGTTCCTCTTGCTGTATAAACAAACACAGCCAGTGTAGCTGCAGGGAGGA
ACTGTGGTGCTGGGGTACAGAGGAAACAATGACTGTAGCCACTACTGCAGATACACTGGAGCTTCAATATCTTAAGAACAACTCACTGACTCGGAATGTCAATATTTCTGTCTTTAAAA
CACAATCAAACCATTCTTTTGTTTCTAACACCAAAAAATTTCCACGTCCTGTCAAAAATAGCTTATGTCATTATGTGTATGTATATACATCTTAGTTCAGCAAAACACTATTTTGGCAATAG
ATTTTTTTATAGTAAGAGCTCTAGCAAGTCTCCCCTTTCCCTCTCCCATGTGATTGATTTACAGTTGGTATTTTTGCATGTTTGGTTTAAGGTGTTTACAAGCTGTTTGTGCTGATGATGC
TGAGCTGCAAAGAGTTCAGGGACCAACGCTGTTCAAAACAGGATGCCACAACATTTTATCTGCTCTGCTGCAGGTGGATTTACCTCCCTGAACACAGGGGATGGGGCTTTTGCTTTTC
AACAACAAAAATACAAAATACTCTCTCCTTTGTTCTGGAGAAAGCCAGAACTTTAAATGTGCTGTTGCATTCACACTGTGACTGCATTAGCTAGGATAGTCAGCCAACAATGCTTTTACC
AGATGATGCGGTCAGACACACCAGGTGAATTCTCCCACCGCTGAGTCATCACATTGCCAATCTCGTCCATAGAGGA

The sequence information of MSTRG.673.2 is shown below. The yellow highlighted region indicates the binding site of MSTRG.673.2 to the seed region of miR-128-3p.

Supplementary Sequence Information for WT and Mut

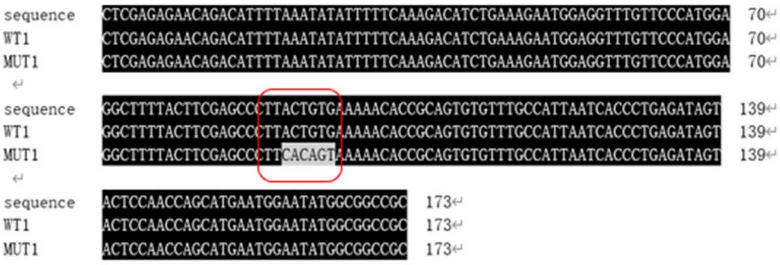

```
sequence  CTCGAGCGTCTGCAATTAAACTGTGAATTCAATTCATTTTCTGGTAATGTGGTTGCTGTTTTGTTGTTT  69
WT2       CTCGAGCGTCTGCAATTAAACTGTGAATTCAATTCATTTTCTGGTAATGTGGTTGCTGTTTTGTTGTTT  69
MUT2      CTCGAGCGTCTGCAATTAACACAGTAATTCAATTCATTTTCTGGTAATGTGGTTGCTGTTTTGTTGTTT  69

sequence  ACAAAGCACATAGACTCGTTATATCCTACTTGGGGACACAAGCCCTCATAGCATTAGAATGAAAATATTT  139
WT2       ACAAAGCACATAGACTCGTTATATCCTACTTGGGGACACAAGCCCTCATAGCATTAGAATGAAAATATTT  139
MUT2      ACAAAGCACATAGACTCGTTATATCCTACTTGGGGACACAAGCCCTCATAGCATTAGAATGAAAATATTT  139

sequence  TCACAATAATTGGAGTGCAATACTGTGATGTCTCAGCTATAGCTACATGAATCAGTGACAGGTAAAATA  208
WT2       TCACAATAATTGGAGTGCAATACTGTGATGTCTCAGCTATAGCTACATGAATCAGTGACAGGTAAAATA  208
MUT2      TCACAATAATTGGAGTGCAATCACAGTATGTCTCAGCTATAGCTACATGAATCAGTGACAGGTAAAATA  208

sequence  TGCAGAGCTGCAATTACATGAAAAATCAACACCTCACTGCTTCGAGGGGCATTCTCATTCAGCGTCCGAA  278
WT2       TGCAGAGCTGCAATTACATGAAAAATCAACACCTCACTGCTTCGAGGGGCATTCTCATTCAGCGTCCGAA  278
MUT2      TGCAGAGCTGCAATTACATGAAAAATCAACACCTCACTGCTTCGAGGGGCATTCTCATTCAGCGTCCGAA  278

sequence  GCCGTGATAAGTGGCTGCAGCTCAAAGAAACATCAGCTTTGCGAGGTCGCTTTGAGCTTGAGGAGCATCT  348
WT2       GCCGTGATAAGTGGCTGCAGCTCAAAGAAACATCAGCTTTGCGAGGTCGCTTTGAGCTTGAGGAGCATCT  348
MUT2      GCCGTGATAAGTGGCTGCAGCTCAAAGAAACATCAGCTTTGCGAGGTCGCTTTGAGCTTGAGGAGCATCT  348

sequence  CCAGAACCGCTGACTCCACACAGTGTGGACAGTGAGGGTGGAAGCATCCCTGCTGGCCAGGCTGGCTCCA  418
WT2       CCAGAACCGCTGACTCCACACAGTGTGGACAGTGAGGGTGGAAGCATCCCTGCTGGCCAGGCTGGCTCCA  418
MUT2      CCAGAACCGCTGACTCCACACAGTGTGGACAGTGAGGGTGGAAGCATCCCTGCTGGCCAGGCTGGCTCCA  418

sequence  CTCTGCACTCAACTGAGATTGAGCCCTCGGAGATCTTGTTGGCAGTGCACAGCTCAAACAGAGGTTGCCC  488
WT2       CTCTGCACTCAACTGAGATTGAGCCCTCGGAGATCTTGTTGGCAGTGCACAGCTCAAACAGAGGTTGCCC  488
MUT2      CTCTGCACTCAACTGAGATTGAGCCCTCGGAGATCTTGTTGGCAGTGCACAGCTCAAACAGAGGTTGCCC  488

sequence  CACTGTGTCCAAAAGCCTGAGCGGCCGC  516
WT2       CACTGTGTCCAAAAGCCTGAGCGGCCGC  516
MUT2      CCACAGTTCCAAAAGCCTGAGCGGCCGC  516

sequence  CTCGAGTACAAAATGAGTAGCATATCTCTCCTGTTCCTCTTGCTGTATAAACAAACACAGCCAGTGTAGC  70
WT3       CTCGAGTACAAAATGAGTAGCATATCTCTCCTGTTCCTCTTGCTGTATAAACAAACACAGCCAGTGTAGC  70
MUT3      CTCGAGTACAAAATGAGTAGCATATCTCTCCTGTTCCTCTTGCTGTATAAACAAACACAGCCAGTGTAGC  70

sequence  TGCAGGGAGGAACTGTGGTGCTGGGGTACAGAGGAAACAATGACTGTAGCCACTACTGCAGATGCGGCC  139
WT3       TGCAGGGAGGAACTGTGGTGCTGGGGTACAGAGGAAACAATGACTGTAGCCACTACTGCAGATGCGGCC  139
MUT3      TGCAGGGAGGACACAGTGTGCTGGGGTACAGAGGAAACAATGACTGTAGCCACTACTGCAGATGCGGCC  139

sequence  GC  141
WT3       GC  141
MUT3      GC  141

sequence  CTCGAGACAAAATACTCTCTCCTTTGTTCTGGAGAAAGCCAGAACTTTAAATGTGCTGTTGCATTCACACTGTGACTGC  79
WT4       CTCGAGACAAAATACTCTCTCCTTTGTTCTGGAGAAAGCCAGAACTTTAAATGTGCTGTTGCATTCACACTGTGACTGC  79
MUT4      CTCGAGACAAAATACTCTCTCCTTTGTTCTGGAGAAAGCCAGAACTTTAAATGTGCTGTTGCATTCACCACAGTACTGC  79

sequence  ATTAGCTAGGATAGTCAGCCAACAATGCTTTTACCAGATGATGCGGTCAGACACACCAGGTGAATTCTCCCAGCGGCCGC  159
WT4       ATTAGCTAGGATAGTCAGCCAACAATGCTTTTACCAGATGATGCGGTCAGACACACCAGGTGAATTCTCCCAGCGGCCGC  159
MUT4      ATTAGCTAGGATAGTCAGCCAACAATGCTTTTACCAGATGATGCGGTCAGACACACCAGGTGAATTCTCCCAGCGGCCGC  159
```

Figure S1. Overexpression and interference efficiency detection of sequencing samples.

A. Detection of miR-128-3p overexpression efficiency B. Detection of miR-128-3p interference efficiency

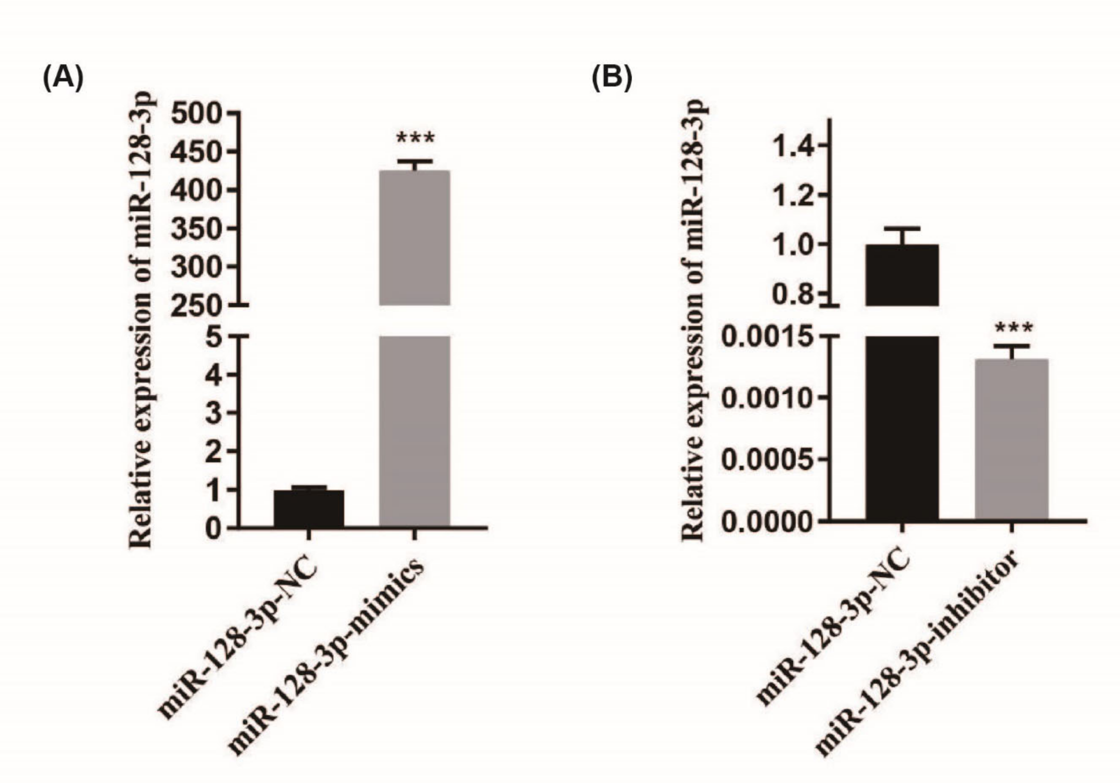

Supplement: Supplementary file 1 [file animals-15-01879-s001.zip › animals-3656224-supplementary.pdf]
